# Two sisters with RSPRY1-related spondyloepimetaphyseal dysplasia

**DOI:** 10.1002/ajmg.a.63601

**Published:** 2024-04-02

**Authors:** Swati Singh, Hitesh Shah, Ashwin Dalal, Anju Shukla, Gandham SriLakshmi Bhavani, Katta M. Girisha

**Affiliations:** 1 Department of Medical Genetics, Kasturba Medical College, Manipal, Manipal Academy of Higher Education, Manipal, India; 2 Department of Pediatric Orthopedics, Kasturba Medical College, Manipal, Manipal Academy of Higher Education, Manipal, India; 3 Diagnostics Division, Centre for DNA Fingerprinting & Diagnostics, Hyderabad, India; 4 Department of Genetics, College of Medicine and Health Sciences, Sultan Qaboos University Muscat, Oman

**Keywords:** Faden-Alkuraya type, *RSPRY1*, spondyloepimetaphyseal dysplasia, vertebral defects

## Abstract

Biallelic variants in *RSPRY1* have been found to result in spondyloepimetaphyseal dysplasia. Two siblings presenting with short stature, facial dysmorphism, progressive vertebral defects, small epiphysis, cupping and fraying of metaphyses, brachydactyly, and short metatarsals harbored a homozygous missense variant c.1652G>A;p.(Cys551Tyr) in the *RSPRY1* gene. The phenotype in our patients resembles spondyloepimetaphyseal dysplasia, Faden-Alkuraya type. Thus, our study provides further evidence to support the association of *RSPRY1* variants with spondyloepimetaphyseal dysplasia. We observed joint dislocation as a novel clinical feature of this condition.

## 1 Introduction

Spondyloepimetaphyseal dysplasia (SEMD), RSPRY1 related (OMIM #616723), also referred to as SEMD, Faden-Alkuraya type, is a rare autosomal recessive disorder. The condition is characterized by short stature, facial dysmorphism, brachydactyly, and intellectual disability. Mild scoliosis, vertebral defects, small epiphysis, cupping, and fraying of metaphyses of tibia and fibula, coxa vara, genu valgum, short metacarpals, short metatarsals, and craniosynostosis have been described in affected individuals (Faden et al., 2015). To date, 10 individuals with SEMD, RSPRY1 type, are reported from 4 unrelated families from Saudi Arabia, Peru, and Turkey (Faden et al., 2015; Simsek-Kiper et al., 2018). Here, we describe the clinical, radio-graphic, and molecular findings of two sisters with RSPRY1-related SEMD from India.

## 2 Methods

Informed consent for medical photography and blood samples for genetic evaluation were obtained from the family members. The study was approved by the institutional ethics committee. Genomic DNA was extracted from the probands and her family from peripheral blood using a QIamp DNA Blood Mini kit (Qiagen). DUO exome sequencing was performed for the similarly affected sisters as described earlier (Girisha et al., 2019). The annotation of the data was performed using ANNOVAR and in-house utility scripts (Kausthubham et al., 2021).

Variant filtering and prioritization were performed using customized in-house scripts. The identified variant was analyzed using in silico pathogenicity prediction tools, CADD phred, REVEL, and M_CAP. Allele frequency of the reported rare variant was estimated using the reference population database, gnomAD (V3.1.2), and our in-house data of 3076 exomes. Sanger sequencing was used for validation and segregation analysis of the rare variant identified in both siblings. The variant is reported using HGVS nomenclature and interpreted as per the relevant recommendations (Pejaver et al., 2022; Richards et al., 2015). The variant is submitted to ClinVar (SCV002061883.2).

## 3 Results

### 3.1 Clinical findings

Two similarly affected sisters from a consanguineous family were evaluated. Both had short stature, facial dysmorphism, lumbar lordosis, and wind-swept deformity.

Proband 1, an 8-year-old girl, was delivered at full term through a lower segment cesarean section in view of a nuchal cord wrapped around her neck. She cried immediately after birth. Her conception was spontaneous, and antenatal scans revealed no notable findings. We do not have anthropometric parameters at birth. However, she did experience neonatal jaundice. Developmentally, she attained head control at 5 months of age, social smile at 3 months, and started to sit without support at 1 year of age. She started walking at 2 years. She was able to speak bisyllables at one and a half years of age and sentences at 2 years. Bilateral lower limb malformation was observed at 4 years of age, which is progressive. She could not walk long distances due to pain. Currently, she is studying in the second grade. No formal IQ assessment was done; however, she responds slowly when questioned or told to follow commands. She was diagnosed with congenital heart disease, patent ductus arteriosus (PDA), and underwent corrective surgery at 3 years of age.

**Figure 1:**
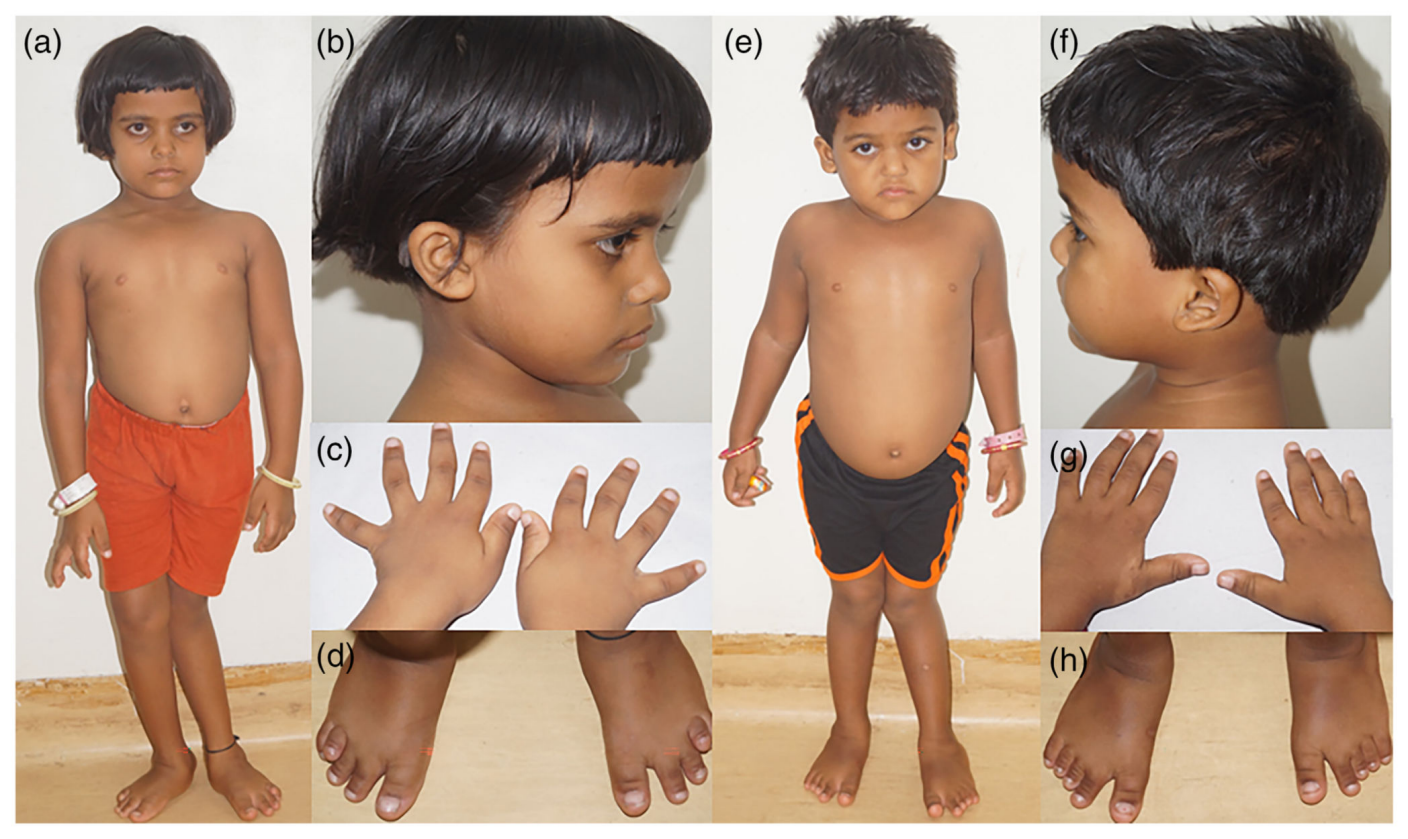
Clinical photographs of Probands 1 (a−d) and 2 (e−h). Both had short stature, wind-swept deformity, and pes planus (a, e). Midface retrusion, frontal bossing, depressed nasal bridge, and short neck can be noted in both (b, f). Both have brachydactyly, clinodactyly (c, g) and broad first toes (d, h). Proband 1 has overriding of the fourth toe (d) and short T4 and T5, whereas Proband 2 has short T3, T4, and T5.

At 8 years, her weight was 17.5 kg (−2.48 SD), height was 98 cm (−5.28 SD), and head circumference was 50 cm (−1.31 SD). She was noted to have a broad forehead, left exotropia with mild ptosis, depressed nasal bridge, high arched palate, low set ears, mid-face retrusion and short neck, brachydactyly, protruded belly, lumbar lordosis, overriding toes, bilateral abducted foot, and wind-swept deformity (Figure 1). She had squint eyes, and the ophthalmologic assessment confirmed myopia in both eyes with mild ptosis in the left eye. Fundus examination was unremarkable.

Proband 2, the younger sibling, was 5 years old when examined. Birth and postnatal periods were unremarkable. Lower limb malformation was observed at the age of 2 years, and it was progressive in nature. She, too, had pain on walking long distances. Her height was 87 cm (−4.53 SD), weight 12.86 kg (−2.61 SD), and head circumference 47 cm (−2.39 SD). She had midface retrusion, a square-shaped face, a depressed nasal bridge, a high arched palate, a short neck, a short thorax, lumbar lordosis, distal joint laxity, and pes planus (Figure 1).

IQ assessment was done for her at 5 years. On Vineland Social Maturity Scale social age was 3 years 1 month and social quotient was 68, indicative of mild deficits in social adaptive functioning. She had generalized tonic−clonic seizures, which lasted for 5 min. The first episode of seizure was noticed at 5 years of age. Since then, five episodes of seizures in 6 months were noted. She was treated with levetiracetam, and her seizures were controlled. Moreover, ophthalmologic evaluation showed myopia in both eyes.

**Figure 2:**
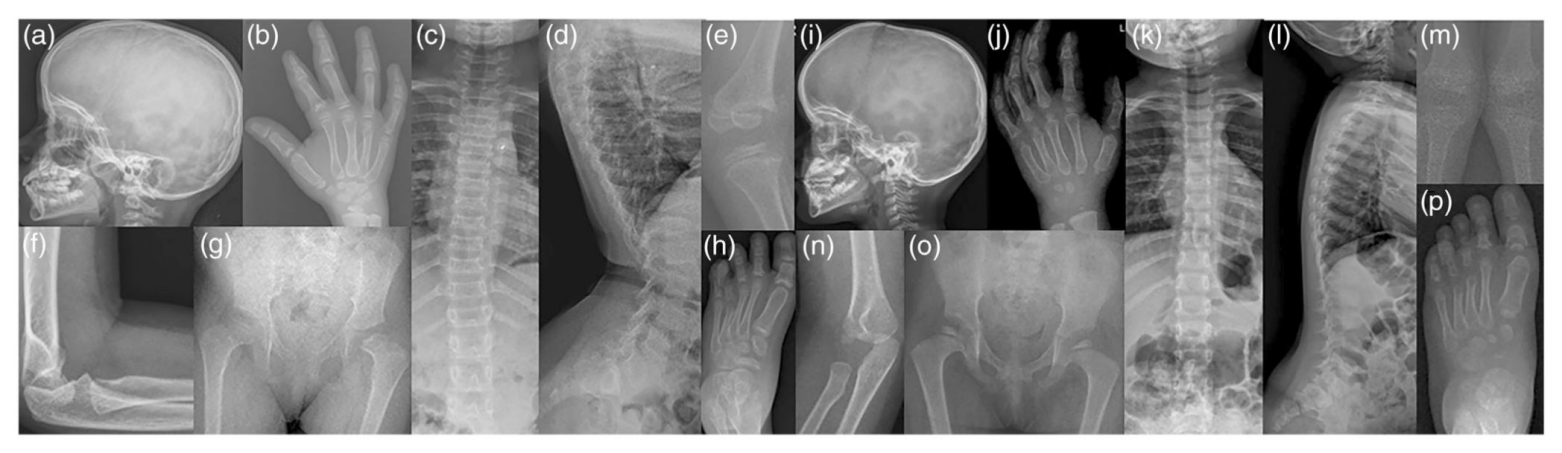
Radiographs of Proband 1 (a−h) at age 8 years and Proband 2 (i−o) at the age of 2 years revealed copper beaten appearance of skull (a, i), clinodactyly of fifth finger, shortened second proximal phalanx, shortened third and fourth metacarpals with small carpals (b) small carpal bones with delayed ossification (j), mild scoliosis (c), and lack of increase in lumbar interpedicular distance (k), lumbar lordosis is evident in both (d, l), along with platyspondyly with anterior beaking of vertebrae (d), small epiphysis, metaphyseal sclerosis, cupping, and fraying of metaphysis at knee joint are exhibited in both (e, m), subluxation of elbow joint (f) and elbow joint dislocation (n), short femoral neck (g, o) with coxa breava (o) and short fourth metatarsal bone is observed in both (h, and p) and short third metatarsal bone (p).

The radiographic evaluation indicated a copper-beaten appearance of a skull, platyspondyly, mild scoliosis, lumbar lordosis, small carpals bones, short femoral neck, short fourth metatarsal bone, wind-swept deformity, small epiphyses, metaphyseal cupping, and fraying in both siblings. In addition, subluxation of the elbow joint in Proband 1 and dislocation and short 3rd metatarsal in Proband 2 were noted (Figure 2).

We could not obtain cranial imaging or electroencephalographic evaluation results for both probands.

### 3.2 Molecular finding

A shared homozygous non-synonymous missense variant at c.1652G>A;p.(Cys551Tyr) in exon 15 of the *RSPRY1* gene (NM_133368.3; NP 588609.1) was identified through exome sequencing in both siblings. This variant is not present in gnomAD (V3.1.2) and our in-house data of 3076 exomes. This variant is predicted to change a cysteine residue to tyrosine in the C3H4C type RING finger domain of the *RSPRY1* gene. Multiple sequence analysis was performed using the Clustal Omega tool (Madeira et al., 2022), and the cysteine amino acid residue was found to be preserved across five vertebrate species (Figure S1). In silico pathogenicity prediction tools comprising CADD phred:29.700, M_CAP: 0.812, REVEL: 0.967 inferred the detected variant to be disease-causing. Sanger sequencing confirmed the carrier status of the parents. Thus, the variant was classified as likely pathogenic [PP3 (strong) + PM2 + PP1 + PP4] based on relevant sequence variant interpretation guidelines (Pejaver et al., 2022; Richards et al., 2015). Furthermore, the alpha missense score for the variant p.(Cys551Tyr) is 0.999, predicting it to be pathogenic as well. However, sufficient information on the structure of this protein is not available (UniProt entry: Q96DX4), including a solved three-dimensional structure or a modeling template. It may be noted that the change of cysteine amino acid residue into a tyrosine at position 551 perturbs the hydrophobic interactions, either in the core of the protein or on the surface. Detailed clinical, radiographic, and molecular findings of both probands are summarized in Table S1.

## 4 Discussion

The first association of the *RSPRY1* gene with skeletal dysplasia was established by Faden et al. (2015). They described a distinct form of SEMD in five patients from two unrelated families. Patients had short stature, intellectual disability, facial dysmorphism, short fourth metatarsals, progressive vertebral defects, and walking difficulties. Subsequently, Kiper et al. reported five additional patients from two unrelated families with a similar phenotype (Simsek-Kiper et al., 2018). Table S1 summarizes the phenotype in all the reported patients to date.

In the present study, we evaluated two sisters who share the phenotype and the disease progression with the previously reported patients. Both had difficulty walking at an early age (2−4 years) and short stature as the presenting symptoms. The severity of vertebral deformities increases with age. We noted subluxation/dislocation of elbow joints in them, which was not reported previously in individuals with RSPRY1-related SEMD.

Congenital heart disease has not been described with this condition earlier. Proband 1 (elder sister) had mild tricuspid regurgitation and Grade I mitral regurgitation. She had undergone surgical repair of her PDA earlier. The exome data analysis also did not reveal any potential pathogenic variants associated with cardiac abnormalities in her. Though PDA could be considered an incidental finding in Proband 1, it is important to note that RSPRY1 is expressed in heart tissue, specifically in the left ventricle and atrial appendage.

*RSPRY1* was first characterized by Waddell et al. RSPRY1 protein is a glycoprotein present in the cytoplasm of skeletal muscle cells. It comprises 576 amino acids. It has B.30/SPRY domain (359−479 aa) and C3HC4-type RING finger domain (526−565 aa). The potential function of this protein is unknown. Although it is likely to be involved in the ubiquitination of target proteins (Waddell et al., 2016) or dysregulation of the FGF signaling pathway (Xie et al., 2020).

In previous studies, two frameshift, one missense, and one canonical splice site variants were reported. In the present study, the biallelic missense variant, c.1652G>A, is detected in exon 15, which is the last exon of the gene (Figure S1). Exon 15 encodes for the C3H4 domain of RSPRY1 protein, which is predicted to be evolutionarily conserved (Figure S1). We speculate alteration at this position impairs the functioning of the RSPRY1 protein, and results in the clinical mani-festations observed in both siblings.

Thus, we describe joint dislocation as a novel clinical feature of RSPRY1-related SEMD while describing a novel causative variant. Due to limited data, additional studies are necessary to functionally characterize the protein and assess its association with genes and pathways related to joint dislocation.

## Supplementary Material

Data S1

Figure S1

Table S1

## Data Availability

The data that support the findings of this study are available from the corresponding author upon reasonable request.
