# Supplementary material for: Two sisters with RSPRY1-related spondyloepimetaphyseal dysplasia
Source: Am J Med Genet A. Author manuscript; Available in PMC 2024 Aug 1. (PMC7616131; doi:10.1002/ajmg.a.63601)
Supplement: Data S1 [file EMS195227-supplement-Data_S1.docx]

**Web resources**

PRIMER 3 v.4.1.0, <http://primer3.ut.ee/>

Ensembl, <https://asia.ensembl.org/index.html>

NCBI, <https://www.ncbi.nlm.nih.gov/>

Mutation Taster, <http://www.mutationtaster.org/>

CADD_Phred, <https://cadd.gs.washington.edu/>

M_CAP, <http://bejerano.stanford.edu/mcap/>

REVEL, <https://genome.ucsc.edu/cgi-bin/hgTrackUi?db=hg19&g=revel>

Online Mendelian Inheritance in Man (OMIM): <https://www.omim.org/>

gnomAD, <https://gnomad.broadinstitute.org/>

HPO, <https://hpo.jax.org/app/>

ClinVar, <https://www.ncbi.nlm.nih.gov/clinvar/>

HGMD, <http://www.hgmd.cf.ac.uk/ac/search.php>

ANNOVAR, <http://annovar.openbioinformatics.org/>
