# Supplementary material for: Two sisters with RSPRY1-related spondyloepimetaphyseal dysplasia
Source: Am J Med Genet A. Author manuscript; Available in PMC 2024 Aug 1. (PMC7616131; doi:10.1002/ajmg.a.63601)
Supplement: Table S1 [file EMS195227-supplement-Table_S1.docx]

| Supplementary table1: Clinical, radiographic and molecular findings in individuals with RSPRY1 related spondyloepimetaphyseal dysplasia | | | | | | | | | | | | |
| --- | --- | --- | --- | --- | --- | --- | --- | --- | --- | --- | --- | --- |
| **Study** | **Faden *et al*., 2015** | | | | | **Kiper *et al*., 2018** | | | | | **Present study** | |
| Family ID | Family 1 | | | | Family 2 | Family 3 | | | | Family 4 | Family 5 | |
| Subject ID | P1 | P2 | P3 | P4 | P5 | P6 | P7 | P8 | P9 | P10 | P11 | P12 |
| **Subject characteristics** | | | | | | | | | | | | |
| Age at examination | 21yrs | 19yrs | 16yrs | 12yrs 5mo | 10yrs 6mo | 11yrs | 16yrs | 18yrs | 35yrs | 9yrs | 8yrs | 5yrs |
| Gender | Female | Female | Female | Male | Male | Male | Female | Male | Male | Male | Female | Female |
| Consanguinity | + | + | + | + | - | + | + | + | + | + | + | + |
| Ethnicity | Saudi Arabia | Saudi Arabia | Saudi Arabia | Saudi Arabia | Peruvian | Turkey | Turkey | Turkey | Turkey | Turkey | Asian Indian | Asian Indian |
| Height in cm (SD) | NA | NA | 100  (-8.2) | NA | NA | 112  (-2.63) | 115  (-4.83) | 142  (-4.3) | 135 (-5.64) | 91  (-2.29) | 98  (-5.28) | 87  (-4.53) |
| Weight in kgs (SD) | NA | NA | 18  (-4.2) | NA | NA | 20  (-1.89) | 36  (-1.47) | 44  (-7.15) | 54  (-7.55) | 13  (-1.56) | 17.5  (-2.48) | 12.86  (-2.61) |
| Head circumference in cm (SD) | NA | NA | (-4.4) | NA | NA | (-0.51) | (-1.41) | (+1.97) | (+2.87) | (-2.92) | 50  (-1.31) | 47  (-2.39) |
| **Clinical features** | | | | | | | | | | | | |
| Progressive walking difficulty | + | + | + | + | + | + | + | + | + | + | + | + |
| Intellectual disability | + | + | + | + | + | + | + | + | + | + | ND | + |
| Delay in motor development | + | + | + | + | + | + | + | + | + | + | + | + |
| Hypertelorism | + | + | + | + | + | + | + | + | + | + | - | - |
| Brachycephaly | NA | NA | + | + | + | + | + | + | + | + | + | + |
| Epicanthal folds | + | + | + | + | + | + | + | - | + | + | - | - |
| Frontal bossing | + | + | + | + | + | + | + | + | + | + | + | + |
| Depressed nasal bridge | + | + | + | + | + | + | + | + | + | + | + | + |
| Full lips | + | + | + | + | + | + | + | + | + | + | - | - |
| Tented upper lip | + | + | + | + | + | + | + | + | + | + | - | - |
| Proptosis | + | + | + | + | + | + | + | + | - | + | - | - |
| Malar hypoplasia | + | + | + | + | - | + | + | + | + | + | + | + |
| Low set ears | - | - | + | + | + | - | - | - | - | - | - | - |
| Ear anomaly | + | + | + | + | + | + | + | + | + | + | - | - |
| Dental anomaly | NA | NA | NA | NA | NA | - | + | - | - | + | - | - |
| Left exotropia | - | - | - | - | - | - | - | - | - | - | + | - |
| High arched palate | - | - | - | - | - | - | - | - | - | - | + | + |
| Cubitus Valgus | - | - | - | - | - | + | + | - | + | + | + | + |
| Clinodactyly | - | - | - | - | - | + | + | + | - | + | + | + |
| Genu valgum | + | - | + | + | + | + | + | + | + | - | + | + |
| Lumbar lordosis | + | - | - | - | + | + | - | + | + | + | + | + |
| Metacarpo-phalangeal joint laxity | NA | NA | NA | NA | NA | + | + | + | + | + | + | + |
| Overriding toes | + | + | + | + | + | - | + | + | + | + | + | + |
| Prominent heels | NA | NA | NA | NA | NA | + | + | + | - | + | - | + |
| Pes planus | NA | NA | NA | NA | NA | + | + | + | - | + | + | + |
| **Radiological features** | | | | | | | | | | | | |
| Craniosynostosis | + | + | + | + | - | - | + | + | + | + | ND | ND |
| Short neck | + | + | + | + | + | - | + | + | + | + | + | + |
| Short trunk | NA | NA | NA | NA | NA | - | + | + | + | - | + | + |
| Kyphoscoliosis | + | + | + | + | + | - | + | + | + | - | + | + |
| Platyspondyly | + | + | + | + | + | + | + | + | + | + | + | + |
| Elbow joint dislocation | - | - | - | - | - | - | - | - | - | - | - | + |
| Metaphyseal dysplasia | + | + | + | + | + | + | + | + | + | + | + | + |
| Epiphyseal dysplasia | + | + | + | + | + | + | + | + | + | + | + | + |
| Hip deformity | + | + | + | + | + | + | + | + | + | + | + | + |
| Knee deformity | + | + | + | + | + | + | + | + | + | + | + | + |
| Delayed bone age | + | + | + | + | + | + | + | + | + | + | + | + |
| Bilateral short 4th metacarpal | + | + | + | + | + | + | + | + | + | + | + | + |
| Small carpal bones | + | + | + | + | + | + | + | + | + | + | + | + |
| Cone shaped epiphyses | + | + | + | + | + | + | + | + | + | + | + | + |
| Short 4th metatarsal | + | + | + | + | + | - | + | + | + | + | + | + |
| Short femoral neck | + | + | + | + | + | + | + | + | + | + | + | + |
| Pectus deformity | - | - | - | - | - | + | + | + | - | + | + | + |
| **Other findings** | | | | | | | | | | | | |
| Cardiac abnormalities | - | - | - | - | - | - | - | - | - | - | + | - |
| Orthopaedic intervention | NA | NA | NA | NA | + | + | + | + | + | - | + | + |
| **Genetic findings in *RSPRY1* (NM_133368.3)** | | | | | | | | | | | | |
| Zygosity | Homozygous | | | | Homozygous | Homozygous | | | | Homozygous | Homozygous | |
| Disease-causing variants | c.1279dupA | | | | c.121G>T | c.377delT | | | | c.516+2T>C | c.1652G>A | |
| Protein change | p.(Thr427AsnfsTer10) | | | | p.(Gly 41Cys) | p.(Ile126LysfsTer20) | | | | - | p.(Cys155Tyr) | |
| Location | Exon 12 | | | | Exon 2 | Exon 3 | | | | Exon 4/ Intron4 | Exon 15 | |
| Variant status | Novel | | | | Novel | Novel | | | | Novel | Novel | |
| ACMG classification | Likely pathogenic (PVS1+ PM2) | | | | Likely pathogenic (PM2+PP3 strong) | Likely pathogenic (PVS1+PM2) | | | | Likely pathogenic (PVS1+PM2) | Likely pathogenic [PM2+PP1+ PP4 + PP3 (strong)] | |

(+): Present; (-): Absent; NA: Not available; ND: Not determined
