# Supplementary material for: Two sisters with RSPRY1-related spondyloepimetaphyseal dysplasia
Source: Am J Med Genet A. Author manuscript; Available in PMC 2024 Aug 1. (PMC7616131; doi:10.1002/ajmg.a.63601)
Supplement: Figure S1 [file EMS195227-supplement-Figure_S1.pdf]

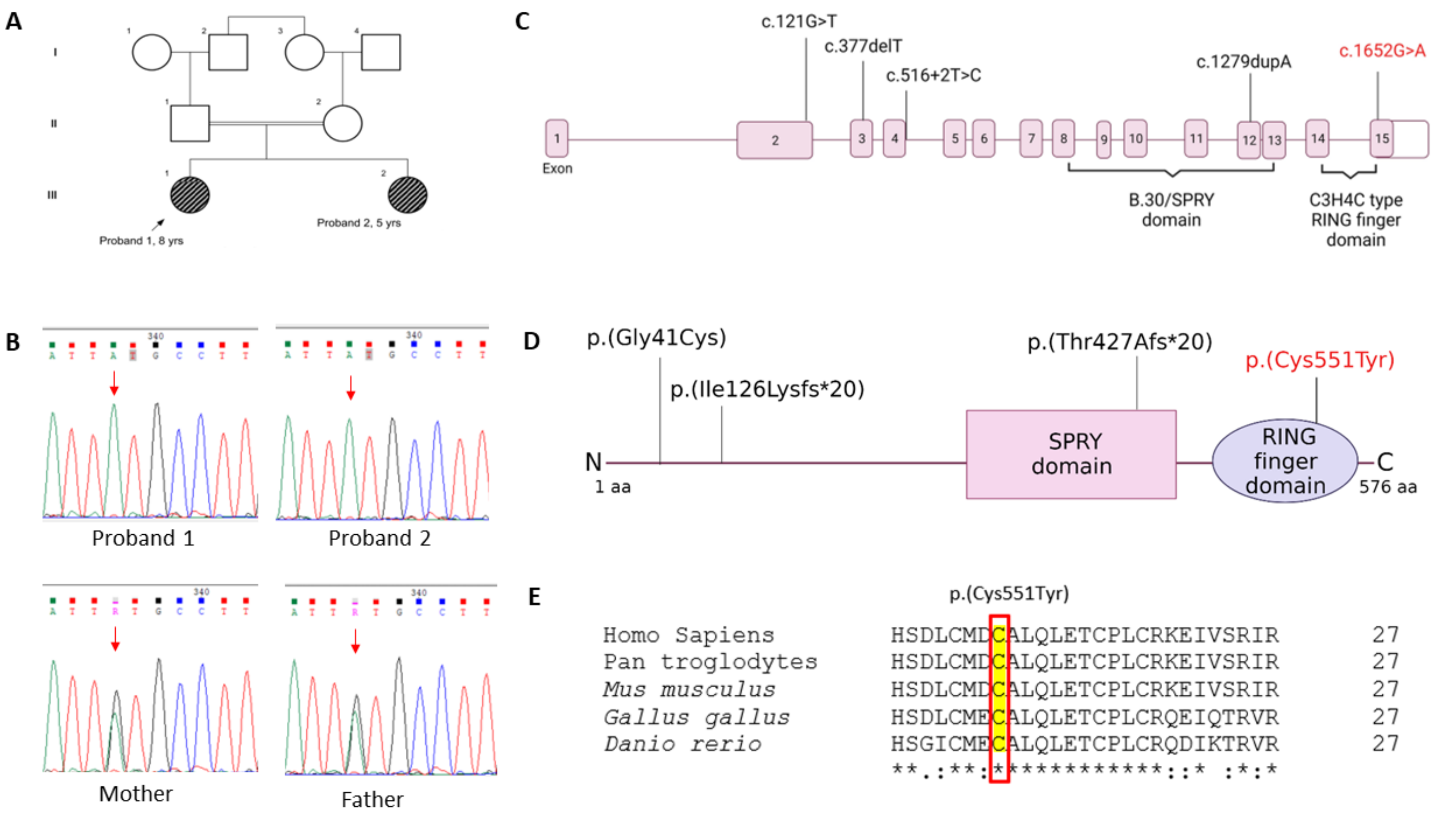

A
I
II
III
Proband 1, 8 yrs
Proband 2, 5 yrs
B
Proband 1
Proband 2
Mother
Father
C
c.121G>T
c.377delT
c.516+2T>C
c.1279dupA
c.1652G>A
Exon
B.30/SPRY domain
C3H4C type RING finger domain
D
p.(Gly41Cys)
p.(Ile126Lysfs*20)
p.(Thr427Afs*20)
p.(Cys551Tyr)
N
1 aa
SPRY domain
RING finger domain
C
576 aa
E
p.(Cys551Tyr)
Homo Sapiens HSDLCMDCALQLETCPLCRKEIVSRIR 27
Pan troglodytes HSDLCMDCALQLETCPLCRKEIVSRIR 27
Mus musculus HSDLCMDCALQLETCPLCRKEIVSRIR 27
Gallus gallus HSDLCMECALQLETCPLCRQEIQTRVR 27
Danio rerio HSGICMECALQLETCPLCRQDIKTRVR 27
.::************::* :*:*
